# Acquired Portosystemic Shunts in Cirrhosis and Portal Vein Thrombosis: A Case Report

**DOI:** 10.7759/cureus.31587

**Published:** 2022-11-16

**Authors:** Anthony Durgham, Steven Tessier, Firas Ido, Santo Longo, Sudip Nanda

**Affiliations:** 1 Department of Gastroenterology and Hepatology, Lewis Katz School of Medicine, Temple University, Philadelphia, USA; 2 Department of Pathology, Lewis Katz School of Medicine, Temple University, Philadelphia, USA; 3 Department of Pulmonary and Critical Care Medicine, St. Luke's University Health Network, Bethlehem, USA; 4 Department of Pathology, St. Luke's University Health Network, Bethlehem, USA; 5 Department of Cardiology, St. Luke's University Health Network, Bethlehem, USA

**Keywords:** hyperammonemia, cirrhosis, portal hypertension, portosystemic shunt, portal vein thrombosis

## Abstract

Acquired portosystemic shunts (PSS) are abnormal blood vessels that develop between the portal vein and systemic circulation as a result of portal hypertension. Recurrent hyperammonemic encephalopathy in our 62-year-old patient with cirrhosis and chronic portal vein thrombosis led to the discovery of an extremely rare and functioning portosystemic shunt (PSS). The PSS connected the inferior mesenteric and left renal veins. Such shunts are considered pathological structures and may require surgical intervention. The large PSS reported herein likely provided decompression of the portal hypertension. The concurrence of portal vein thrombosis clearly precluded any consideration of surgery. Therapeutic management in each instance of these shunts requires a full understanding of their origination, location, and physiologic implications.

## Introduction

Portosystemic shunts (PSS) are abnormal venous conduits that direct the hepatic portal blood flow to the systemic circulation, bypassing normal flow to the liver. These shunts are classified as either congenital or acquired. Congenital PSS are present at birth, with symptoms typically presenting during early childhood. Acquired PSS are those that manifest later in life, either via the opening of a previously nonfunctional congenital PSS or possibly via neoangiogenesis [1]. The initial clinical expression of acquired shunts in adults is usually the result of portal hypertension [1,2]. With normal portal pressures, the majority of PSS remain asymptomatic. The most prominent among the various causes of portal hypertension are cirrhosis, severe congestive heart failure, and portal vein occlusions [3]. The acquisition of blood flow through the PSS may provide compensatory relief of the portal hypertension [2]. In the instance of portal vein thrombosis, the return of normal portal pressure may be attempted by vascular recanalization of the obstructing thrombus, referred to as a cavernoma [4]. In the absence of this physiologic restoration, an alternate shunting of blood through a PSS may develop due to the persistence of portal hypertension.

Our case of a very large PSS is a rare mesorenal shunt connecting the inferior mesenteric vein and the left renal vein (IMV-RV). This patient’s shunt remained open and functional for several years. The presence of chronic portal vein thrombosis and gastrointestinal bleeding due to mucosal arteriovenous malformations (AVMs) favored nonsurgical management.

## Case presentation

**Figure 1:**
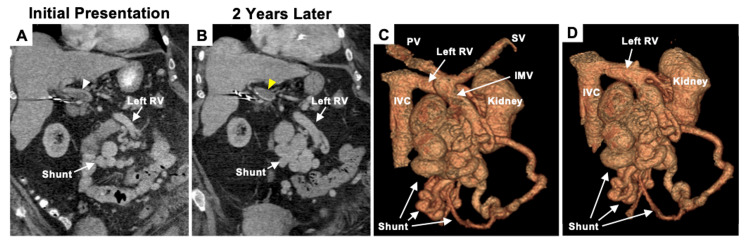
Chronic portal vein thrombosis and 3D reconstruction of the mesorenal shunt. Coronal CT of the abdomen/pelvis without contrast showing (A) nonocclusive portal vein thrombosis (white arrowhead), (B) completely occlusive portal vein thrombosis two years later, and a cross-section of the developing mesorenal shunt. (C) 3D reconstruction of the IMV-RV shunt. (D) 3D reconstruction of the IMV-RV shunt with the IMV, PV, and SV removed showing the shunt connecting with the left RV. CT: computed tomography; IVC: inferior vena cava; PV: portal vein; RV: renal vein, IMV: inferior mesenteric vein; SV: splenic vein

A 62-year-old female with a history of cirrhosis secondary to nonalcoholic steatohepatitis (Child-Pugh score 7, class B), chronic kidney disease, and chronic diastolic heart failure presented to the emergency department (ED) with left upper quadrant pain that radiated to the left scapula, as well as lethargy. Her cirrhosis was confirmed with a liver biopsy 12 years prior, which showed a grade 2, stage 4 cirrhotic liver with macrovesicular steatosis. Aside from the detection of left upper quadrant tenderness and mild splenomegaly, the physical examination was unremarkable. Blood pressure was 130/80 mmHg, heart rate 88 bpm, and respiratory rate 14 bpm. Complete blood count was notable for a hemoglobin of 11.1 g/dL and platelet count of 104,000/mL. Without any evidence of hematologic disorders or coagulopathy, the thrombocytopenia was ascribed to congestive splenomegaly secondary to portal hypertension. Several outpatient visits for her portal hypertension included an unremarkable oesophagogastroduodenoscopy (OGD). Abdominal computed tomography (CT) disclosed a large subtotal occlusion of the portal vein by a bland portal vein thrombus (Figure 1A). This thrombus was located in the main portal vein without extension to the superior mesenteric or splenic veins. Incidentally, a varicocele of the left ovarian vein and a venous shunt between the inferior mesenteric and left renal veins (IMV-RV) was also demonstrated (Figure 1A). The patient was evaluated by gastroenterology who recommended conservative management with observation.

In the ensuing two years, the patient and her family noted recurrent and worsening episodes of mental confusion and generalized weakness. These symptoms prompted measurement of her serum ammonia level, which was elevated to 59 mmol/L (Child-Pugh score 7, class B). Her mental status was improved with lactulose and rifaximin. She was also found to be anemic (hemoglobin: 8.1 g/dL). OGD and colonoscopy revealed gastric antral vascular ectasia (GAVE) and ascending colonic AVMs, which were subsequently cauterized. The patient was discharged home on lactulose (30 mL, twice daily) and rifaximin (550 mg, twice daily). The serum ammonia level one month later was normal (26 mmol/L) on the prescribed regimen.

The patient remained asymptomatic for an additional two years before she presented to the ED with generalized weakness and constipation for 10 days. After four units of packed red blood cells (RBCs), a hemoglobin of 4.9 g/dL rose to 7.5 g/dL. Repeat OGD and colonoscopy were unrevealing. Push enteroscopy demonstrated multiple AVMs in the third and fourth portions of the duodenum, which were refractory to clipping and argon plasma coagulation (APC). On abdominal CT, the known chronic portal vein thrombus was now completely occlusive, and the IMV-RV shunt had considerably increased in size (Figure 1B). Subcutaneous octreotide (50 mcg, twice daily) was prescribed, and the patient was discharged home with instructions for immediate outpatient follow-up with gastroenterology.

One month later, the patient presented with hyperammonemia (172 mmol/L), encephalopathy, and worsening pitting edema of the bilateral extremities (Child-Pugh score 8, class B). Hemoglobin was 6.6 g/dL. Lactulose, rifaximin, and enemas failed to lower her ammonia levels. She required numerous blood transfusions for presumed gastrointestinal blood loss and anemia of chronic disease. During this hospitalization, the patient received continuous veno-venous hemofiltration for a decline in renal function. The family elected to prioritize patient comfort due to persistent critical illness and refractory encephalopathy. After several days, the patient died in hospice care.

## Discussion

Our patient with a 12-year history of cirrhosis presented with persistent portal vein thrombosis. The management protocols for persistent portal vein thrombosis are most extensively discussed in the presence of cirrhotic liver disease. The recommended treatment guidelines of the American College of Gastroenterology for chronic portal vein thrombosis due to liver disease include anticoagulation with warfarin or low-molecular-weight heparin for six months, although management is generally individualized [5]. These recommendations hold constant for patients with complications such as inherited thrombophilia, propagation of the thrombus, or evidence of bowel ischemia [5]. However, while anticoagulation therapy may result in increased recanalization rate and decreased thrombosis recurrence rates, no mortality benefit has been described [6]. Thrombolysis has been proposed as a therapy for portal thrombosis. However, in comparison to anticoagulation, thrombolysis has demonstrated increased morbidity and mortality [4].

**Figure 2:**
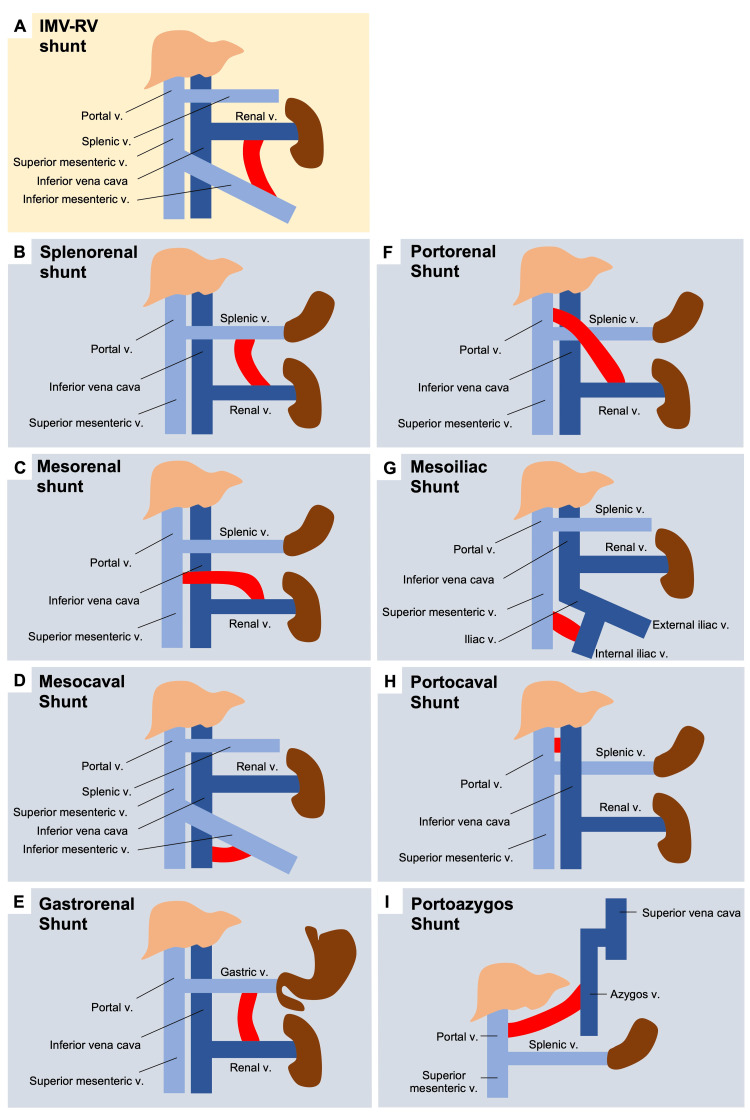
Diagrams of portosystemic shunt variants. (A) Mesorenal shunt in our patient connecting the inferior mesenteric vein and renal vein. Shunts between (B) the splenic vein and the renal vein [7], (C) the superior mesenteric vein and the renal vein [8], (D) the inferior mesenteric vein and the inferior vena cava [9], (E) the left gastric vein and the renal vein [10], (F) the renal vein and the portal vein [11], (G) the superior mesenteric vein and the iliac vein [12], (H) the portal vein and the inferior vena cava [13], and (I) the azygos vein and the portal vein [14]. Dark blue veins: systemic circulation; light blue veins: portal circulation

In the absence of portal vein recanalization, chronic portal vein thrombosis may lead to the recruitment of PSS, with notable variants described in Figure 2.

There is little data regarding the management of these shunts following portal vein thrombosis. The management of these PSS is challenging given the rarity of this scenario. Guidelines for the management of PSS recommend shunt ligation using metallic coils in patients with refractory hepatic encephalopathy [2]. However, these recommendations are directed at PSS in the absence of portal vein thrombosis. Franzoni et al. described the rare occurrence of both a large splenorenal shunt and portal vein thrombosis in the presence of several other smaller PSS. Embolic closure of the largest shunt resulted in marked improvement of hepatic encephalopathy. Shunt embolization was only possible in this case due to the presence of other smaller shunts [15].

## Conclusions

We report a case illustrating the importance of a full assessment and understanding of the origination, location, and physiologic implications of a newly discovered PSS. This applies to patients with or without chronic liver disease, portal hypertension, and/or portal vein thrombosis. In our patient with chronic liver disease and portal vein thrombosis, the spontaneous and extremely rare IMV-RV PSS was silent and totally unsuspected. While this PSS very likely aggravated our patient’s hyperammonemia, it also likely provided some degree of decompression of the portal hypertension. The extremely rare location of her IMV-RV shunt has been documented only in a few case reports. The presence of a PSS in the setting of portal vein thrombosis is a similarly rare occurrence, which must be managed on a case-by-case basis.

## References

[1] Rajesh S, Philips CA, Ahamed R, Abduljaleel JK, Nair DC, Augustine P (2021). Friend or foe? Spontaneous portosystemic shunts in cirrhosis-current understanding and future prospects. Can J Gastroenterol Hepatol.

[2] Leite AF, Mota A Jr, Chagas-Neto FA, Teixeira SR, Elias Junior J, Muglia VF (2016). Acquired portosystemic collaterals: anatomy and imaging. Radiol Bras.

[3] Simonetto DA, Liu M, Kamath PS (2019). Portal hypertension and related complications: diagnosis and management. Mayo Clin Proc.

[4] Northup PG, Garcia-Pagan JC, Garcia-Tsao G (2021). Vascular liver disorders, portal vein thrombosis, and procedural bleeding in patients with liver disease: 2020 practice guidance by the American Association for the Study of Liver Diseases. Hepatology.

[5] Simonetto DA, Singal AK, Garcia-Tsao G, Caldwell SH, Ahn J, Kamath PS (2020). ACG clinical guideline: disorders of the hepatic and mesenteric circulation. Am J Gastroenterol.

[6] Wu M, Schuster M, Tadros M (2019). Update on management of portal vein thrombosis and the role of novel anticoagulants. J Clin Transl Hepatol.

[7] Iannello S, Libertini L, Martini R (1999). A large spontaneous splenorenal shunt in a patient with liver cirrhosis and uncomplicated portal hypertension. Dig Dis.

[8] Kikuchi H, Noguchi M, Akashi H, Noda S (2001). Spontaneous shunt between the superior mesenteric vein and the right renal vein caused by portal hypertension. J Urol.

[9] Kakizawa H, Toyota N, Hieda M (2011). Portal-systemic shunt between the inferior mesenteric vein and inferior vena cava in a patient with hepatic encephalopathy: successful occlusion by balloon-occluded retrograde transvenous obliteration. Hiroshima J Med Sci.

[10] Dick E, Watkinson A (1999). A spontaneous portosystemic shunt from the left gastric vein to the left renal vein caused by portal hypertension. AJR Am J Roentgenol.

[11] Sabol Pušić M, Budimir I, Dorosulić Z, Ostrički B, Nikolić M, Lovrenčić Prpić G, Sreter KB (2017). Portal systemic shunt between the hepatic portal vein and right renal vein in a patient with multifocal hepatocellular carcinoma: case report. J Clin Ultrasound.

[12] Ali S, Stolpen AH, Schmidt WN (2010). Portosystemic encephalopathy due to mesoiliac shunt in a patient without cirrhosis. J Clin Gastroenterol.

[13] Palvanov A, Marder RL, Siegel D (2016). Asymptomatic intrahepatic portosystemic venous shunt: to treat or not to treat?. Int J Angiol.

[14] Gebrael J, Yu H, Hyslop WB (2013). Spontaneous portoazygos shunt in a patient with portal hypertension. J Radiol Case Rep.

[15] Franzoni Lde C, de Carvalho FC, Garzon RG (2014). Embolization of splenorenal shunt associated to portal vein thrombosis and hepatic encephalopathy. World J Gastroenterol.

